# Moving Auto-Correlation Window Approach for Heart Rate Estimation in Ballistocardiography Extracted by Mattress-Integrated Accelerometers

**DOI:** 10.3390/s20185438

**Published:** 2020-09-22

**Authors:** Marco Laurino, Danilo Menicucci, Angelo Gemignani, Nicola Carbonaro, Alessandro Tognetti

**Affiliations:** 1 National Research Council, Institute of Clinical Physiology, 56124 Pisa, Italy; laurino@ifc.cnr.it (M.L.); angelo.gemignani@unipi.it (A.G.); 2 Department of Surgical, Medical and Molecular Pathology and Critical Care Medicine, University of Pisa, 56124 Pisa, Italy; danilo.menicucci@unipi.it; 3 Department of Information Engineering, University of Pisa, 56124 Pisa, Italy; nicola.carbonaro@unipi.it

**Keywords:** ballistocardiogram, accelerometer, sensors, unobtrusive cardiac monitoring, smart bed, auto-correlation, heart rate, heart rate variability

## Abstract

Continuous heart monitoring is essential for early detection and diagnosis of cardiovascular diseases, which are key factors for the evaluation of health status in the general population. Therefore, in the future, it will be increasingly important to develop unobtrusive and transparent cardiac monitoring technologies for the population. The possible approaches are the development of wearable technologies or the integration of sensors in daily-life objects. We developed a smart bed for monitoring cardiorespiratory functions during the night or in the case of continuous monitoring of bedridden patients. The mattress includes three accelerometers for the estimation of the ballistocardiogram (BCG). BCG signal is generated due to the vibrational activity of the body in response to the cardiac ejection of blood. BCG is a promising technique but is usually replaced by electrocardiogram due to the difficulty involved in detecting and processing the BCG signals. In this work, we describe a new algorithm for heart parameter extraction from the BCG signal, based on a moving auto-correlation sliding-window. We tested our method on a group of volunteers with the simultaneous co-registration of electrocardiogram (ECG) using a single-lead configuration. Comparisons with ECG reference signals indicated that the algorithm performed satisfactorily. The results presented demonstrate that valuable cardiac information can be obtained from the BCG signal extracted by low cost sensors integrated in the mattress. Thus, a continuous unobtrusive heart-monitoring through a smart bed is now feasible.

## 1. Introduction

Health systems around the world are facing significant challenges related to the growth in the aging population and the increasing number of people with chronic and infectious diseases. The recent outbreak of the COVID-19 disease has pointed out the strong need for effective telehealth platforms able to remotely improve the quality of patients’ healthcare while maintaining low costs for the services provided [1,2]. The current trend is to integrate data obtained at the clinical routine with daily life real-world data captured by unobtrusive sensor technology to enable better monitoring and management of patients [3,4,5]. In this context, continuous cardiac monitoring is essential for early detection and diagnosis of cardiovascular diseases, which are key factors for the evaluation of health status in the general population. Therefore, it is important to develop unobtrusive and transparent cardiac monitoring technologies for the general population.

The possible approaches are the development of wearable technologies or the integration of sensors in daily-life objects [6]. As described in our previous works reported in [7,8,9], for non-invasive sleep quality and cardio-respiratory assessment, we developed the Smart-Bed prototype in the L.A.I.D project (Linking Automation to artificial Intelligence for revealing sleep Dysfunctions [10]). The Smart-Bed is based on a single mattress made by Materassificio Montalese (Pistoia, Italy) and was designed to detect the subject’s motion and position, physiological signals (heart rate and breathing rate) and environmental parameters (sound intensity, relative humidity, room temperature and luminosity). In [8], we demonstrated that the Smart-Bed performs well compared to polysomnography and correctly classifies the behavioural conditions useful to create an objective sleep quality index.

In this work, we describe the potential of using our Smart-Bed prototype as a non-invasive cardiac monitoring system able to detect-at home during the night or 24 h for bedridden patients-subjects’ heartbeats and to consequently extract information about heart rate (HR) and heart rate variability (HRV). In particular, to measure the cardiac activity, we used the information of one of the three accelerometers integrated into the mattress for the estimation of the ballistocardiogram (BCG). The BCG signal is generated by the vibrational activity of the body in response to the cardiac ejection of blood [11]. BCG is a promising technique but is usually replaced by electrocardiogram (ECG) due to the complexity associated with the signal processing (for an exhaustive overview of BCG technologies and algorithms, please refer to the review paper from Sadek et al. [12]).

We have developed a new algorithm for heart rate extraction from the BCG signal, based on a moving auto-correlation sliding-window applied on the signal measured from the accelerometer. We have tested our method on a group of 10 healthy volunteers with the simultaneous co-registration of ECG using a single-lead configuration. Comparisons with ECG reference signals indicated that the proposed algorithm performed satisfactorily. The results presented demonstrate that valuable cardiac information can be obtained from the BCG signal obtained by a low-cost sensor (i.e., the accelerometer) integrated in the mattress.

Our algorithm employs a time-domain approach, but, unlike the main previous time-domain approaches [12], it does not find a specific BCG waveform which intrinsically changes depending on the subject, the position and the experimental context. Instead, our approach is based on the hypothesis that two successive heartbeats in the BCG signal have undefined but similar waveforms. Thus, our algorithm estimates, through auto-correlation sliding windows, the signal periodicity. This allows us to overcome the problems of long-range non-stationarity and non-linearity of the BCG signal. With respect to previous works employing auto-correlation approaches such as [13,14,15,16,17], we obtained a greater time resolution of BCG-based tachogram (1 Hz) that allows extracting information not only of HR but also about HRV.

## 2. Materials and Methods

### 2.1. Smart-Bed Prototype

**Figure 1:**
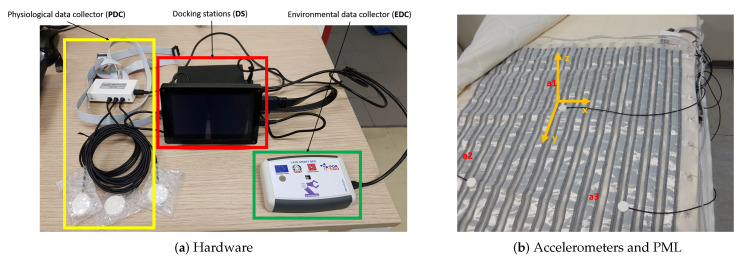
Smart-Bed prototype: (**a**) Functional blocks of the Smart-Bed prototype. Docking station (DS) is in the red frame, physiological data collector (PDC) in the yellow frame and environmental data collector (EDC) in the green frame; (**b**) Position of the three accelerometers (*a*_1_, *a*_2_ and *a*_3_) over the pressure mapping layer (PML).

As described in [7,8] and as shown in Figure 1a, the Smart-Bed prototype includes a Docking station (DS), a physiological data collector (PDC) and an environmental data collector (EDC). The PDC and EDC are wired to DS via USB serial communication. The DS is a Microsoft Windows 10-based system. The DS is equipped with tailored software for: (i) managing PDC and EDC, (ii) processing the signals and parameters from PDC/EDC, (iii) storing the collected data, and (iv) extracting the sleep and environmental quality indices. The PDC is equipped with a pressure mapping layer (PML) and a set of three tri-axial accelerometers (Figure 1b).

The PML, based on a piezoresistive textile applied onto the foam layer below the mattress top cover, is designed to detect the distribution of pressures when a subject is lying on the bed and to consequently detect the subject’s position and movements. To extract the BCG signal, the PDC is equipped with three micropower digital accelerometer (ADXL362, Analog Device Inc., Norwood, MA, USA). The main specifications of the ADXL362 are reported in Table 1.

As shown in Figure 1b, the accelerometers are placed over the PML just below the mattress top cover. The Smart-Bed simultaneously collects the signals of the three accelerometers (*a*_1_, *a*_2_ and *a*_3_) placed in different positions. Accelerometer signals are acquired using 256 Hz sampling rate.

For this study, we have considered the contribution of the accelerometer $a_{1}$ placed in the central area of the mattress corresponding to the position of the chest of the subject over the mattress. The axes of accelerometer $a_{1}$ were oriented as shown in Figure 1b.

### 2.2. Data Collection and Pre-Processing

We tested our algorithm with the data recorded from 10 healthy volunteers (4 female, 6 male, mean age: 25.8 years, mean BMI: 24.9 kg $m^{-2}$). All participants gave their informed consent for inclusion before they participated in the study. The study was conducted in accordance with the Declaration of Helsinki, and the protocol was approved by the Ethics Committee of the University of Pisa (0102206/2019).

Data were collected when participants were physically relaxed laying over the sensing mattress in the supine position. During the experiments, we collected simultaneously BCG (from accelerometer $a_{1}$) and ECG signals. For ECG data collection, a multi-channel polygraph (Be-Micro holter, EBNeuro spa, Florence, Italy) was used. ECG is acquired using 256 Hz sampling rate and it is synchronized with BCG acquisition. During the BCG and ECG collection, participants were asked to be still, silent and unmoving for about 10 min.

We consideredthe BCG signal to be accelerometric signal correspondent to the longitudinal axes in the head-to-toe direction of the subject over the mattress (Y-axis in Figure 1b). The artefacts of ECG and BCG signals, due to the subject movements at the beginning and the end of recording, were detected by visual inspection and they were manually removed. The minimum length of the resulting recordings was 120 s. The lead II of ECG was used as gold-standard reference for heart rate estimation, the RR-intervals were estimated from ECG by using the Pan-Tompkins’ algorithm for R-peak detection [18]. The estimated RR-interval signal was linearly interpolated with 1 Hz sampling frequency to obtain a constant sample interval, in this way we could compare the RR-interval signals estimated from ECG and BCG aligned to the same sample interval. The outliers of RR-interval signals were removed by filtering with Hampel filter [19] considering a sliding window of 10 s and a difference of 3 standard deviations at least.

To reduce the components due to respiratory activity or high-frequency noise and enhance the cardiac activity on BCG, the BCG signal was band-passed with a Chebyshev II filter, in the frequency range of 0.5–20 Hz with 60 dB stop-band attenuation. The cut-off frequencies correspond to the significant cardiac information content in the BCG spectrum [20]. Successively, the BCG signal was smoothed by using a Savitzky-Golay finite impulse response filter [21] of polynomial order 3 and frame length 10 msec.

### 2.3. BCG Processing Algorithm

**Figure 2:**
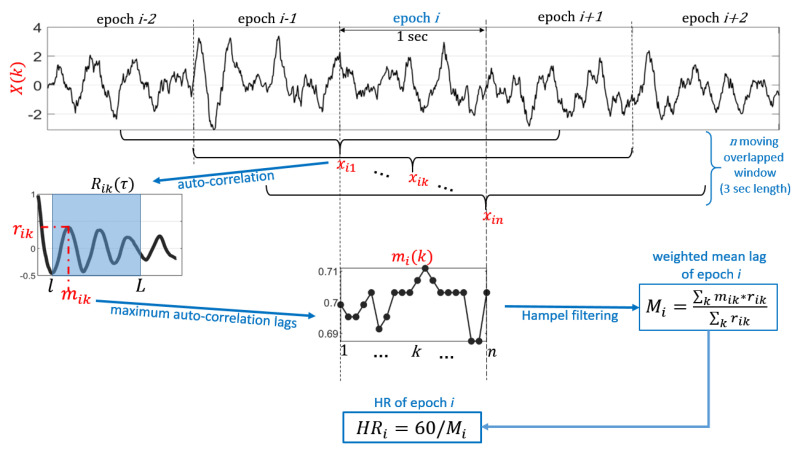
Figure algorithm.

In the proposed algorithm (Figure 2), the heart rate was calculated by analyzing the BCG signal in time domain, with an approach not sensitive to the shape of the signal but adaptive to the changes of BCG waveform morphology.

The discrete time BCG signal X(t) was splitted in epochs of 1 s time duration. For each *i* epoch, *n* moving overlapped windows ($x_{i1}\left(t\right),x_{i2}\left(t\right),\cdots x_{ik}\left(t\right),\cdots x_{in}\left(t\right)$) of 3 s duration (*T*) centered within the *i* epoch were extracted from BCG signal X(t). The time shifting between consecutive overlapped windows was 50 msec. Considering a mean HR between 40 and 100 bpm during resting activities or sleep, the period of BCG waveform is between 1.5 to 0.6 s, respectively. The length of 3 s of moving overlapped windows $x_{ik}$ was chosen in order to include in each time windows at least two consecutive BCG waveform periods, independently on the phase of the waveform.

Following the linear detrending of each time window $x_{ik}\left(t\right)$, the auto-correlation sequence $R_{ik}\left(\tau \right)$ of each $x_{ik}\left(t\right)$ was estimated as a function of time lag τ with a minimum lag (*l*) of 0.6 s and a maximum lag (*L*) of 1.5 s, as they correspond to the minimum and maximum HR which can be estimated (i.e., 40–100 bpm). $R_{ik}\left(\tau \right)$ was normalized so that the auto-correlation at zero lag was equal to 1:

(1) $$R_{ik}\left(\tau \right)=\frac{\sum_{t=0}^{T-\tau -1}\left(x_{ik}\left(t\right)-\mu \right)\left(x_{ik}\left(t-\tau \right)-\mu \right)}{R_{ik}\left(0\right)}$$

where l≤τ≤L; μ and *T* are the mean value and total length of the time window $x_{ik}\left(t\right)$, respectively.

Then, we extracted the the temporal location $m_{ik}$ and the amplitude $r_{ik}$ of absolute maximum of $R_{ik}\left(\tau \right)$. The local maxima of $R_{ik}\left(\tau \right)$ are detected by considering a difference threshold of 0.4 between each maximum and its surrounding values. The temporal location of the absolute maximum ($m_{ik}$) corresponds to the temporal periodicity of *i*-th epoch, and thus it is an estimation of beat-to-beat interval (BBI) at time *k* of the *i*-th epoch of the discrete BCG signal X(t). After obtaining the sequence of maximum auto-correlation lags ($m_{ik}$), we removed the outliers by Hampel filtering with a sliding window of 10 moving windows and a difference of 3 standard deviations at least.

For each epoch *i* of BCG signal X(t), we estimated the weighted arithmetic mean ($M_{i}$) of the maximum auto-correlation lag sequence $m_{ik}$ considering as relative weights the correspondent auto-correlation peak value ($r_{ik}$), as follows:

(2) $$M_{i}=\frac{\sum_{\bar{k}=k}^{k+n}m_{i\bar{k}}\times r_{i\bar{k}}}{\sum_{\bar{k}=k}^{k+n}r_{i\bar{k}}}$$

The obtained mean of the maximum auto-correlation lags ($M_{i}$) was an estimation of RR time interval at *i* epoch. Hence, the heart rate referred to *i* epoch ($HR_{i}$) was calculated as:

(3) $$HR_{i}=\frac{60}{M_{i}}$$

Finally, to minimize the number of false estimations, the $HR_{i}$ outliers were removed by Hampel filtering with a sliding window of 10 epochs and a difference of 3 standard deviations at least. The possible missing values of $HR_{i}$ sequence were replaced by using a piecewise cubic spline interpolation.

In the proposed algorithm, it is possible to modify the parameters according to specific needs or study conditions. For example, the temporal length of each epoch can be increased (e.g., 2 or 5 s as shown in appendix) if a fine temporal resolution of the tachogram is not needed. Also, the minimum (l) and maximum lags (L) of the auto-correlation function can be modulated in order to search the HR in different intervals.

### 2.4. Validation Procedure

The HR estimated from BCG was evaluated by comparison with the reference HR from ECG. The comparison was based on the mean absolute error (MAE), and root mean squared error (RMSE), Pearson’s correlation coefficients (R), and correspondent *p*-values evaluated subject by subject.

To evaluate the overall inter-rater assessment, the Bland-Altman plot of HR sequences pooled over all the subjects was performed, and the following parameters were estimated: reproducibility coefficient (RPC), coefficient of variation expressed as a percentage (CV), root mean squared error (RMSE) and squared Pearson r-value ($r^{2}$).

To evaluate the capability of our algorithm to extract also information about HRV for each subject, we compared the following HR/HRV parameters [22] in time and frequency domains estimated from both BCG and ECG: mean RR interval (mean RR), standard deviation of RR intervals (std RR), standard deviation of differences between adjacent RR intervals (stdDRR), root mean square of differences of successive RR intervals (rmsDRR), number of successive difference of intervals which differ by more of 50 msec divided by the total number of RR intervals (pnn50), absolute power of the low-frequency band (0.04–0.15 Hz, HF), absolute power of the high-frequency band (0.15–0.4 Hz, LF) and the ratio of absolute powers between low-frequency and high-frequency bands (LF/HF). We statistically compared the difference of HR/HRV indices between BCG and ECG by means of paired *t*-tests and Pearson’s correlations.

The data management, the algorithm implementation and validation analyses were carried out using Matlab (R2020a, Mathworks, Natick, MA, USA).

## 3. Results

Considering all the 10 healthy volunteers, we have analyzed 3733 heartbeats from BCG that have been compared with the ones estimated from ECG. The results are evaluated by considering the temporal length of each epoch of 1 s, which is the most challenging condition. In Appendix A, we show results about our BCG method with the longest lengths of the temporal epochs (2 and 5 s).

**Figure 3:**
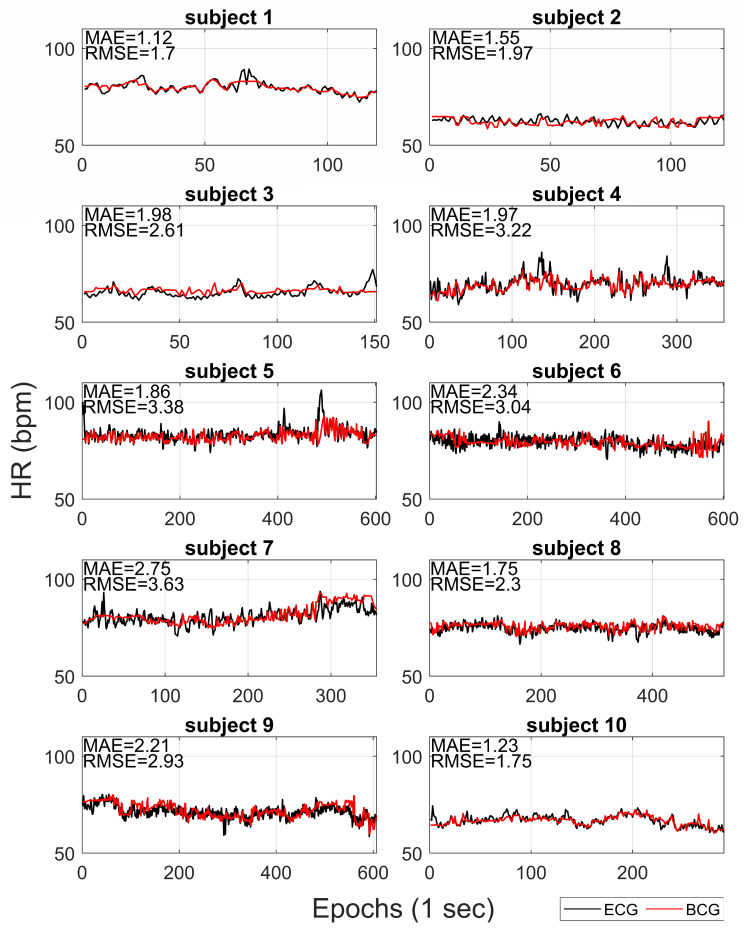
For each subject, the HR extracted from BCG (red lines) and HR from ECG (black lines) are reported. The correspondent mean absolute errors (MAE) and root mean squared errors (RMSE) are shown.

Figure 3 compares the HR extracted from BCG with reference HR from ECG for each subject. The mean absolute error (MAE) and the root mean squared error (RMSE) are also reported on each recording. On average, the MAE and RMSE were 1.88 bpm (ranges from 1.12 to 2.75) and 2.65 bpm (ranges from 1.70 to 3.63), respectively.

**Figure 4:**
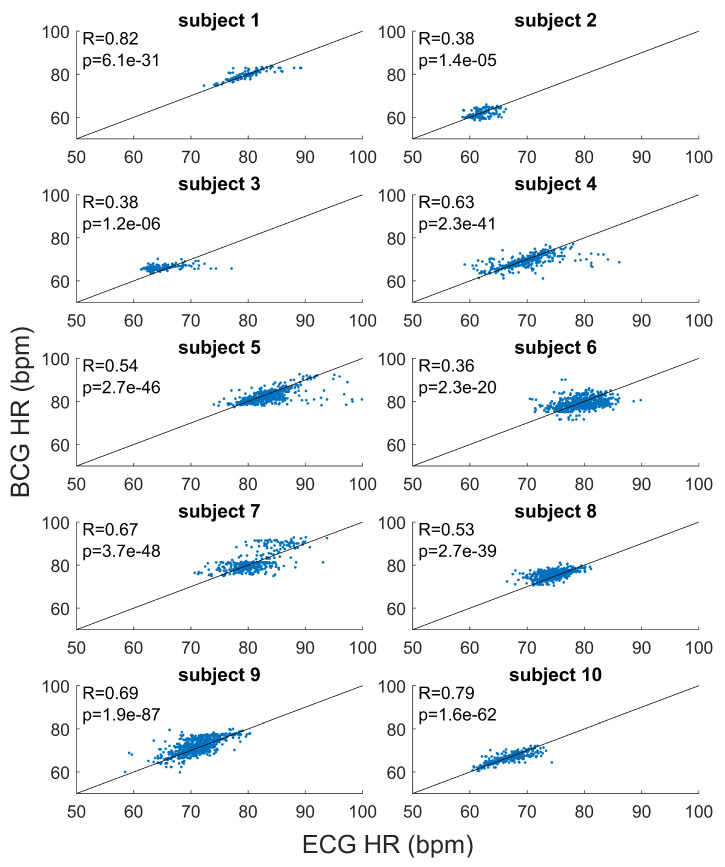
For each subject, the scatter plots and linear regression lines for HR estimated from BCG and ECG are reported. The correspondent coefficient of regression (R) and *p*-values (*p*) are shown.

The results of the subject by subject correlation analysis are shown in Figure 4. Positive linear correlations were found between the HR estimated from BCG and HR from ECG, for all the recordings (*p*-values <0.001). On average, the Pearson’s correlation coefficient was 0.58 (ranges from 0.37 to 0.82).

**Figure 5:**
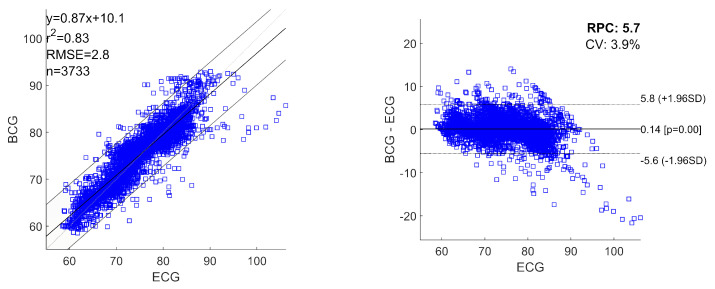
Linear regression plot and Bland-Altman plot of all the analysed heartbeats for ECG and BCG. The heartbeats are pooled over all the recordings. In the plots are reported: linear regression equation, squared Pearson r-value ($r^{2}$), root mean squared error (RMSE), number of heartbeats analysed (n), reproducibility coefficient (RPC) and coefficient of variation expressed as a percentage (CV). The lines of mean of difference between ECG and BCG, and 95% limits of agreement are reported.

Figure 5 shows the linear regression plot and Bland-Altman plots for all the heartbeats estimated with ECG and BCG, considering the heartbeats pooled over all the recordings. The obtained regression equation is:

(4) y=0.87∗x+10.1

where *x* and *y* are the HRs obtained from ECG and BCG, respectively. For pooled linear regression, the squared Pearson r-value ($r^{2}$) was 0.83 and RMSE was 2.8 bpm. The Bland-Altman analysis reported a RPC of 5.7 and a CV of 3.9%. The mean difference of simultaneously recorded heartbeats for ECG and BCG is 0.14 bpm (*p*-values <0.001) with 95% limits of agreement (±1.96SD) in +5.8 bpm and −5.6 bpm.

**Figure 6:**
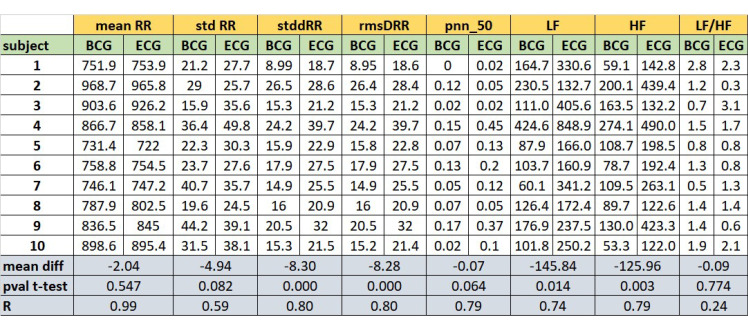
Comparative table of HR and HRV parameters between the estimation from ECG and BCG: mean RR interval (meanRR, in msec), standard deviation of RR intervals (stdRR, in msec), standard deviation of differences between adjacent RR intervals (stdDRR, in msec), root mean square of differences of successive RR intervals (rmsDRR, in msec), number of successive difference of intervals which differ by more of 50 msec divided by the total number of RR intervals (pnn50, adimensional number), absolute power of the low-frequency band (0.04–0.15 Hz-LF, in dB), absolute power of the high-frequency band (0.15–0.4 Hz-HF, in dB) and the ratio of absolute powers between low-frequency and high-frequency bands (LF/HF, adimensional number). The mean differences (mDs), the *p*-values (*p*-values) of mean differences and Pearson’s correlation coefficients (R) between the BCG and ECG estimations for all parameters are also reported.

Figure 6 reports the comparison of some HR and HRV parameters between the ECG and BCG estimations. For each parameter, the table shows its estimation evaluated for each subject. Considering the estimations subject by subject, Figure 6 reports the mean difference between ECG and BCG estimations (mDs), the *p*-values of mDs evaluated by paired *t*-tests and the Pearson’s correlation coefficients (R).

The considered parameters were: mean RR interval (meanRR), standard deviation of RR intervals (stdRR), standard deviation of differences between adjacent RR intervals (stdDRR), root mean square of differences of successive RR intervals (rmsDRR), number of successive difference of intervals which differ by more of 50 msec divided by the total number of RR intervals (pnn50), absolute power of the low-frequency band (0.04–0.15 Hz, LF), absolute power of the high-frequency band (0.15–0.4 Hz, HF) and the ratio of absolute powers between low-frequency and high-frequency bands (LF/HF, adimensional number). No significant differences (*p*-values >0.05) are found in the comparisons of the ECG and BCG estimations for the following parameters: meanRR, stdRR, pnn50 and LF/HF. The linear correlations coefficients are high (at least R>0.59) for all the parameters, except for LF/HF ratio.

**Figure 7:**
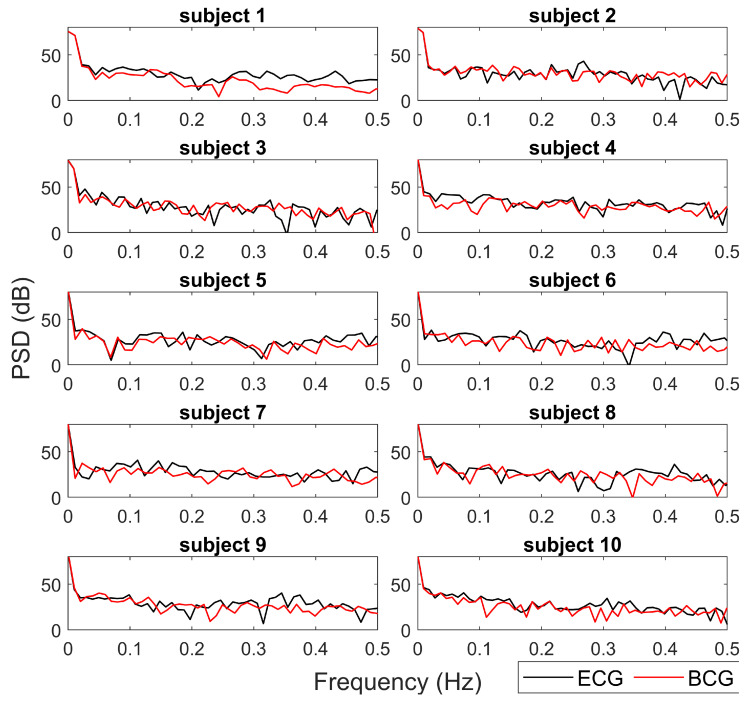
For each subject, the Power Spectrum Densities (PSD) of tachograms from BCG (red lines) and ECG (black lines) are reported. The PSD are expressed in dB.

Figure 7 reports the Power Spectrum Density (PSD) estimated from the tachograms from BCG (red lines) and ECG (black lines) for a qualitative comparison. The spectra obtained from the two different data sources (ECG and BCG) show similar trends and values in all HRV frequency bands. In most cases (subjects 1, 5, 6, 8, 9), the main deviations between the ECG-PSD and BCG-PSD are in high frequency bands (>0.15Hz).

## 4. Discussion

In this study, we describe a new BCG-based algorithm for non-invasive cardiac monitoring. The proposed algorithm allows estimating the HR and HRV information from BCG for subjects lying on the Smart-Bed prototype. Our approach is time-domain and it consists of a moving auto-correlation sliding-window applied on the signal measured from an accelerometer integrated in a mattress.

The performance of our method is satisfactory given the low-cost and low-invasiveness of the hardware solution adopted for BCG signal collection. On average across the subjects, our method obtains an MAE of 1.88 bpm (ranges from 1.12 to 2.75) and an RMSE of 2.65 bpm (ranges from 1.70 to 3.63). For all recordings, the tachogram estimated from BCG is highly significantly correlated (*p*-values < 0.001) with the tachogram from ECG (mean Pearson’s correlation coefficient of 0.58). From Bland-Altman analysis, the RPC and CV assumes low values as well as 5.7 and 3.9%, respectively.

Several of the existing time-domain approaches for HR estimation from BCG signals are able to detect the temporal locations of the heartbeats through the analysis of specific BCG waveforms or template temporal features (template-based methods) [23,24,25]. Nevertheless, the recognition of a specific BCG waveform is challenging because the signal waveform has large variability depending on the subjects, their position on the mattress and the experimental conditions. Hence, our algorithm is based on the idea that two successive heartbeats in a BCG signal have an undefined (i.e., due to subject variability, subject position on the mattress, sensor location) but similar waveform. Our algorithm estimates the periodicity of the signal through auto-correlation sliding windows, thus overcoming the problems of long-range non-stationarity and non-linearity of the BCG signal [11,12].

One of the main distinctive features of our approach is the possibility to estimate the tachogram from the BCG signal (collected by a single low-cost accelerometer) with a high time resolution (i.e., 1 Hz). In this way, our method allows us to extract information not only of HR but also about HRV. The proposed algorithm results able to robustly estimate the mean HR and slow trends for each recording, but it was less efficient in fast variation in HR. In Figure 3 and Figure 4, it is possible to note how the average HR is accurately and precisely estimated with BCG for each subject. Also, the slow fluctuations and changes in HR are well-estimated and tracked by BCG (e.g., see the slow trends in subjects 7 and 9 in Figure 3). Otherwise, the most evident differences between HR estimations from ECG and BCG are found in the case of faster deviations of HR in ECG. Also in Figure 5, the most significant deviations of HR from ECG and BCG were in the case of high values of HR due to fast changes in tachogram. This low-band pass behaviour of our algorithm is expected because of the presence of low-band pass filters (Savitzky-Golay and Hampel) necessary for compensating the artefacts and noise in the BCG signal. The differences in spectrum composition between tachograms obtained from ECG and BCG are also highlighted by the difference in spectral HRV parameters (LF and HR in Figure 6). On average, the BCG-based estimations over a subject of LF and HF significantly differs from the ECG-based estimation. However, the LF and HF estimated from BCG are highly correlated with the estimations from ECG. In addition, also the PSD estimated from the tachograms (see Figure 7) shows similar qualitative trends between ECG and BCG for all the subjects. The main differences in the PSD are the high-frequency band, showing that the proposed algorithm allows a robust spectral evaluation of cardiac signal in particular consider the low frequency activity.

Concerning the evaluated HRV parameters, except for LF/HF ratio, the estimations performed from BCG with our approach are highly correlated with the corresponded gold standard estimation with ECG. Likely, the linear relationship of LF/HR between BCG and ECG is altered by specific subject estimations (subjects 2, 3 and 9). Future works will investigate also the non-linear domain features of HRV estimated with the proposed algorithm.

In summary, the present work demonstrates that HR and HRV parameters can be robustly estimated with the developed Smart-Bed prototype equipped with a single low-cost accelerometric sensor placed over the mattress, by using the proposed novel ballistocardiography algorithm.

## 5. Conclusions

In this work, we described a new algorithm for the estimation of HR and HRV information from a BCG signal extracted by a single low-cost accelerometers integrated in a mattress. The proposed method is based on a time-domain approach and does not consider a specific waveform of the BCG signal.

The major limitations of this study are: (i) the small sample size of subjects, (ii) the short duration of the recordings, (iii) the specific position (supine) of the subjects during experimental sessions, (iv) the manual detection and exclusion of the artefacts from the recordings and (v) limited number of HRV parameters to compare the robustness of HRV estimation. Notwithstanding these limitations, our work suggests that our method is able to robustly estimate the tachogram and HRV information in time and spectral domains from a BCG signal collected with a low-cost accelerometer placed over a mattress.

Future studies will be oriented to a more extensive testing phase with more subjects and in real-world contexts. Starting from the results obtained, we will develop data fusion approaches exploiting the information from the complete set of accelerometers and the pressure sensing matrix. Indeed, by knowing the position and movements of the subjects on the mattress, it will be possible to compensate for the artifacts or to automatically select artifact-free sections of the BCG signal (e.g., when the pressure sensing matrix detects no movement). A further development could be to fuse accelerometer and pressure-sensing matrix signals to estimate breathing rate and to detect relevant physiological events such as sleep apnea which is relevant for several pathological conditions.

## Figures and Tables

**Table 1:** Accelerometer specifications.

Specification	Value
Number of axes	3
Range	±2 g
Supply voltage	3.3 V
Sensitivity	1 mg/LSB
Raw data noise level	175μg/$\sqrt{\mathrm{Hz}}$ (ultralow noise mode)
Bit resolution	12 bits
Sampling frequency	256 Hz

**Figure A1:**
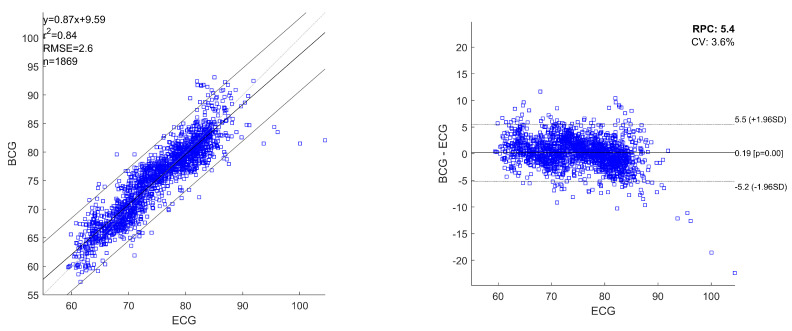
Linear regression plot and Bland-Altman plot of the analysed heartbeats for ECG and BCG considering the temporal lengths of 2 s for the epochs. The heartbeats are pooled over all the recordings. In the plots are reported: linear regression equation, squared Pearson r-value ($r^{2}$), root mean squared error (RMSE), number of heartbeats analysed (n), reproducibility coefficient (RPC) and coefficient of variation expressed as a percentage (CV). The lines of mean of difference between ECG and BCG, and 95% limits of agreement are reported.

**Figure A2:**
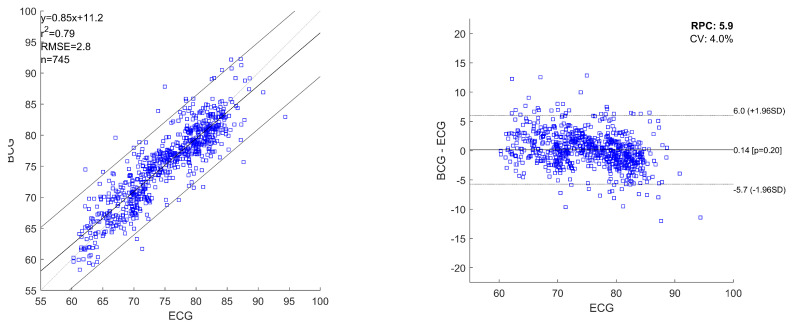
Linear regression plot and Bland-Altman plot of the the analysed heartbeats for ECG and BCG considering the temporal lengths of 5 s for the epochs. The heartbeats are pooled over all the recordings. In the plots are reported: linear regression equation, squared Pearson r-value ($r^{2}$), root mean squared error (RMSE), number of heartbeats analysed (n), reproducibility coefficient (RPC) and coefficient of variation expressed as a percentage (CV). The lines of mean of difference between ECG and BCG, and 95% limits of agreement are reported.

## Abbreviations

The following abbreviations are used in this manuscript:

BCG	Ballistocardiography
ECG	Electrocardiography
HR	Heart Rate
HRV	Heart Rate Variability
DS	Docking Station
PDC	Physiological Data Collector
EDC	Environmental Data Collector
PML	Pressure Mapping Layer
RPC	ReProducibility Coefficient
CV	Coefficient of Variation
RMSE	Root Mean Squared Error
MAE	Mean Absolute Error

## Appendix A. Results for Longest Temporal Lengths of the Epochs

The main results of this work are obtained with a temporal length of each epoch of 1 s. The time length of 1 s allows estimating the HR and HRV parameters with a temporal resolution almost identical to the ECG estimation. However, the short temporal length of 1 s is a challenging condition because it is more sensitive to noise. In this appendix, we show brief results about the application of our BCG method with the longest temporal lengths of the epochs (2 and 5 s). These results show that our methods can estimate the HR and HRV parameters with a short temporal resolution of 1 s without a substantial loss of performance.

Figure A1 shows the linear regression plot and Bland-Altman plots for all the heartbeats estimated with ECG and BCG, considering the heartbeats pooled over all the recordings and the temporal lengths of 2 s for the epochs. The obtained regression equation is:

(A1) y=0.87∗x+9.59

The variables *x* and *y* are the HRs obtained from ECG and BCG, respectively. For pooled linear regression with 2 s length of epochs, the squared Pearson r-value ($r^{2}$) was 0.84 and RMSE was 2.6 bpm. The Bland-Altman analysis reported a RPC of 5.4 and CV of 3.6%. The mean difference of simultaneously recorded heartbeats for ECG and BCG is 0.19 bpm (*p*-values <0.001) with 95% limits of agreement (±1.96SD) in +5.5 bpm and −5.2 bpm.

Figure A2 shows the linear regression plot and Bland-Altman plots for all the heartbeats estimated with ECG and BCG, considering the heartbeats pooled over all the recordings and the temporal lengths of 5 s for the epochs. The obtained regression equation is:

(A2) y=0.85∗x+11.20

The variables *x* and *y* are the HRs obtained from ECG and BCG, respectively. For pooled linear regression with 5 s length of epochs, the squared Pearson r-value ($r^{2}$) was 0.79 and RMSE was 2.8 bpm. The Bland-Altman analysis reported a RPC of 5.9 and CV of 4.0%. There is not a significant difference of simultaneously recorded heartbeats for ECG and BCG (*p*-values =0.20) and the 95% limits of agreement (±1.96SD) are +6.50 bpm and −5.7 bpm.
